# 1303. Pelvic Acute Hematogenous Osteomyelitis in Children, from 2012-2020

**DOI:** 10.1093/ofid/ofad500.1142

**Published:** 2023-11-27

**Authors:** Adriana Sarmiento Clemente, J Chase McNeil, Kristina G Hulten, Jesus G Vallejo, Sheldon L Kaplan

**Affiliations:** Arkansas Children's Hospital, Little Rock, Arkansas; Baylor College of Medicine, Houston, Texas; Baylor College of Medicine, Houston, Texas; Baylor College of Medicine, Houston, Texas; Baylor College of Medicine, Houston, Texas

## Abstract

**Background:**

Pelvic osteomyelitis has been reported to account for 1-11% of acute hematogenous osteomyelitis (AHO) cases in children. Higher complication rates and need for longer antibiotic courses have been suggested by experts, but recent data is lacking. In our retrospective study from 2012-2020, pelvis and tibia were the most frequent AHO sites (24.6% of cases each). We describe the clinical course of children admitted to Texas Children’s Hospital (TCH) with pelvic AHO.

**Methods:**

A retrospective review of patients with diagnosis of AHO admitted to TCH from Jan. 2012 - Dec. 2020 was conducted. Patients 6 mo-< 19 years-old and with ≤ 14 days of symptoms on admission were eligible. Patients with sickle cell disease or those immunocompromised were excluded. Wilcoxon rank sum test for continuous variables and Fisher’s exact for categorical variables were performed using STATA 17.

**Results:**

103 cases of pelvic AHO were compared to 315 cases of non-pelvic AHO (Table 1). The pelvis areas involved were reviewed (Fig. 1). Fever was present in 91 cases of pelvis AHO (88.4%), localized pain in 78 (75.7%) and refusal to bear weight in 64 (62.1%). All patients with pelvic AHO had an abnormal C reactive protein ( > 0.5 mg/dl).

Patients with pelvic AHO had overall similar microbiology (Fig. 2), length of stay and complication rates as non-pelvic AHO. Pelvic AHO patients had pyomyositis more often (29.1 vs 9.2%) and bone abscess less often (22.3 vs 46.3%). Need for bone debridement or bone abscess drainage was less common (17.5 vs 51.1%), but muscle abscess drainage (16.5 vs 5.1%) and percutaneous bone biopsy (22.3 vs 13.7%) were more common in pelvic AHO. 22 cases of pelvic AHO had bone biopsy only, leading to identification of etiology in 27% of them with no complications.

Patients were treated with antibiotics for a similar time (median 41 vs. 38 days). 21 patients with pelvic AHO (20.4%) were treated for ≤ 30 days with no complications (2 were lost to follow up).

**Figure:**
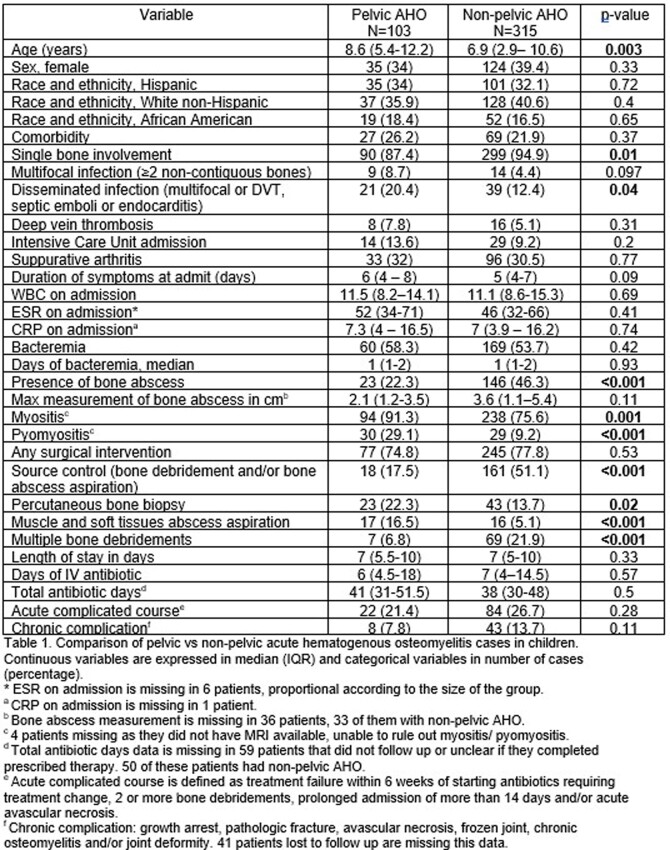

**Figure 1.**

**Figure:**
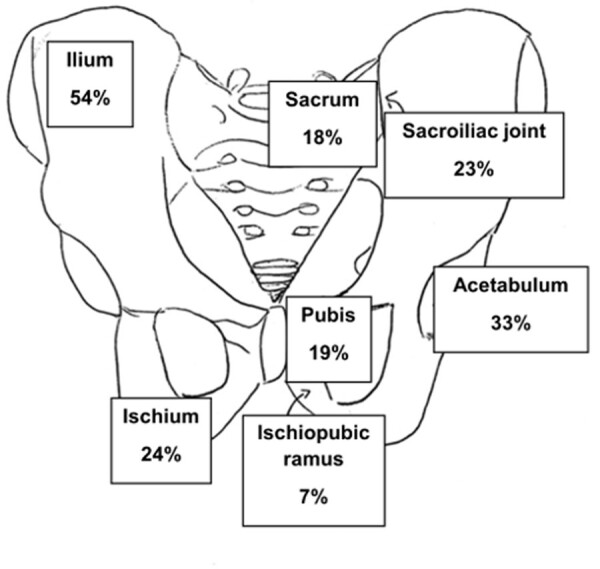

Percentage of each area of pelvic bone involved in patients with pelvic AHO. Patients may have more than one area involved.

**Figure 2.**

**Figure:**
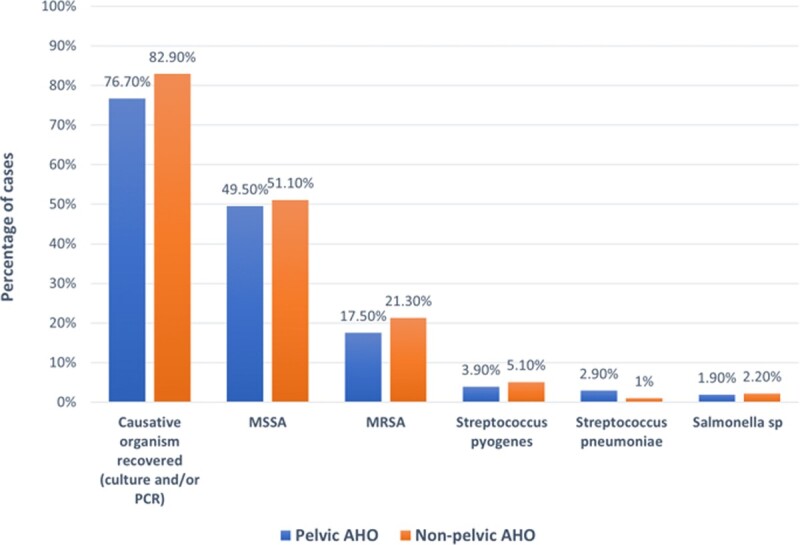

Causative organism recovery and etiology of pediatric cases with pelvic and non-pelvic acute hematogenous osteomyelitis, Texas Children’s Hospital 2012-2022.

**Conclusion:**

Pelvic AHO in children may be more frequent than previously reported but is not associated with more chronic complications. MRI use can aid in the prompt identification of associated pyomyositis and timely intervention. A conservative approach of bone biopsy in cases with a small bone abscess and no pyomyositis should be considered. 4 weeks of therapy may be sufficient.

**Disclosures:**

**J. Chase McNeil, MD**, Allergan: Grant/Research Support|Nabriva Therapeutics: Grant/Research Support **Kristina G. Hulten, PhD**, Pfizer: Grant/Research Support **Sheldon L. Kaplan, MD**, MeMed: Grant/Research Support|Pfizer: Grant/Research Support|Pfizer: Honoraria

